# Microencapsulated foods as a functional delivery vehicle for omega-3 fatty acids: a pilot study

**DOI:** 10.1186/1550-2783-6-12

**Published:** 2009-05-29

**Authors:** Conrad P Earnest, Molly K Hammar, Monica Munsey, Catherine R Mikus, Robert M David, J Alexander Bralley, Timothy S Church

**Affiliations:** 1 Pennington Biomedical Research Center, Baton Rouge, Louisiana, USA; 2 Rush University, Chicago, Illinois, USA; 3 Wake Forest University, Winston-Salem, North Carolina, USA; 4 Metametrix Clinical Laboratory, Norcross, Georgia, USA

## Abstract

It is well established that the ingestion of the omega-3 (N3) fatty acids docosahexaenoic acid (DHA) and eicosapentaenoic acid (EPA) positively benefit a variety of health indices. Despite these benefits the actual intake of fish derived N3 is relatively small in the United States. The primary aim of our study was to examine a technology capable of delivering omega-3 fatty acids in common foods via microencapsulation (MicroN3) in young, healthy, active participants who are at low risk for cardiovascular disease. Accordingly, we randomized 20 participants (25.4 ± 6.2 y; 73.4 ± 5.1 kg) to receive the double blind delivery of a placebo-matched breakfast meal (~2093 kJ) containing MicroN3 (450–550 mg EPA/DHA) during a 2-week pilot trial. Overall, we observed no differences in overall dietary macronutrient intake other than the N3 delivery during our treatment regimen. Post-test ANOVA analysis showed a significant elevation in mean (SE) plasma DHA (91.18 ± 9.3 vs. 125.58 ± 11.3 *u*mol/L; P < 0.05) and a reduction in triacylglycerols (89.89 ± 12.8 vs. 80.78 ± 10.4 mg/dL; P < 0.05) accompanying the MicroN3 treatment that was significantly different from placebo (P < 0.05). In post study interviews, participants reported that the ingested food was well-tolerated, contained no fish taste, odor or gastrointestinal distress accompanying treatment. The use of MicroN3 foods provides a novel delivery system for the delivery of essential fatty acids. Our study demonstrates that MicroN3 foods promote the absorption of essential N3, demonstrate bioactivity within 2 weeks of ingestion and are well tolerated in young, active participants who are at low risk for cardiovascular disease.

## Introduction

Consumption of oily fish or oils rich in the omega-3 fatty acids (N3) docosahexaenoic acid (DHA) and eicosapentaenoic acid (EPA) are well established for their role in supporting cardiovascular health [1-3]. While the mechanisms surrounding the cardioprotective effects of EPA and DHA are complex, they can be broadly categorized into modulations of cardiac function (including antiarrhythmic effects), hemodynamics (cardiac mechanics), arterial endothelial function, and the modulation of lipids, in particular triacylglycerols [2,4].

Despite these benefits the actual intake of fish derived N3 is relatively small in the United States whereby total N3 accounts for 1.6 g/d (0.7% of energy intake). Of this, about 1.4 g/d is plant derived α-linolenic acid (ALA), whereas only 0.1 to 0.2 g/d comes from EPA and DHA [2]. Supplementation with N3 capsules is an option; however, gastrointestinal disturbances and fish odor often contribute to low compliance. Moreover, little research has been performed on younger, healthy and active participants at low risk for cardiovascular disease. It is intuitive, however, that early prophylaxis via the ingestion of N3 while young is beneficial for long term health. The aim of our investigation was to perform a pilot trial to test the feasibility of using foods fortified with microencapsulated fish oil (MicroN3) to deliver a beneficial daily amount of EPA and DHA to individuals not regularly consuming fish or N3 supplement products.

## Methods

We obtained written informed consent from 20 participants (12 men, and 8 women; 20–70 y) in generally good health, who agreed to maintain their current diet and exercise habits (3–5 days/wk) during the trial. Participants were excluded if their BMI was <18.5 or >34.9. We also excluded candidates currently taking an N3 supplement or eating fish > 1×/wk. Participants were randomized equally to a treatment or placebo group after completing all questionnaires inclusive of food frequency measurements. On days 0 and 15 blood was collected for analysis (see below). On days 1–14, participants reported to our kitchen to consume a breakfast meal (~2093 kJ). The treatment breakfast of foods containing MicroN3 (MEG-3™; Ocean Nutrition, Nova Scotia, Canada) included: milk, yogurt, and bread products including tortillas and sliced bread. All of the products we used in our study were "finished goods" products available in grocery stores in the United States and Canada. Thus, each product was made with the MEG-3 ingredient all ready in place. We did not use the MEG-3 product as a powder that was mixed into foods. A list of foods currently available can be found at . We also incorporated brown eggs from hens fed flaxseed as hens are able to efficiently convert the ALA derived from flax to DHA [5]. Total EPA/DHA ranged from 450–500 mg/meal. Individuals randomized to the placebo group received macronutrient-matched meals. This study protocol was approved by the Institutional Review Board at The Cooper Institute, Dallas, TX, USA.

Primary outcomes included plasma concentrations of the fatty acids EPA and DHA, which are typically associated with cardiovascular health [2-4]. All plasma fatty acid analysis was completed in one batch at Metametrix Clinical Laboratory (Norcross, GA, USA) using gas chromatography/mass spectrometry [6]. We obtained 12 hour fasting blood samples from all study participants on days 0 and 15. For plasma samples, we drew one 7 mL EDTA (lavender) tube, inverted the tube ~10 times and centrifuged the sample immediately for 15 minutes. We then transferred 3 ml of plasma to a transfer tube and kept the sample frozen until we performed our analysis in batch.

Plasma fatty acids were analyzed in duplicate using gas chromatography/mass spectrometry (GC/MS). Sample preparation consists of a methyl esterification reaction followed by liquid/liquid extraction prior to analysis. To a 16 × 100 mm glass screw top tube, 2 mL of internal standard solution was added to 200 μL of plasma. Samples were vortex mixed followed by a 1.5 mL addition of reaction solution (1:3 v/v, acetyl chloride:iso-octane). Sample tubes were capped, vortex mixed, and placed in a heat block at 100°C for 1 hour. Samples were cooled and neutralized with 4 mL of potassium carbonate (100 mg/L in H2O). The samples were vortex mixed and centrifuged at 3500 RPMs for 10 minutes. The top layer of the biphasic sample solution was extracted into amber auto-sampler vials and loaded on instrument. The samples were analyzed using an Agilent 6890N GC with autosampler and an Agilent 5973N mass spectrometer. The analytical separation was performed on a HP-23 (Cis/Trans FAME capillary column) 60 m × 0.25 mm × 0.25 mm film thickness. The instrumental and data analysis were performed using MSD Chem Station.

We also examined plasma lipids and hepatorenal function, with a particular interest in triacylglycerols as a surrogate clinical feature reflective of the physiologic activity of N3 supplementation. In order to examine dietary intake, we used the FIAS system (version 3.9, 2000) developed at the Human Nutrition Center, University of Texas Health Science Center School of Public Health. One reason we have selected the FIAS is that it is linked with the Pyramid Serving Database (PSDB). The USDA food codes generated after the analysis of the dietary recalls in FIAS are linked to the PSDB to determine the number of servings of each major food groups consumed. This database was developed to analyze the number of servings of each of the Food Guide Pyramid's major food groups and the amounts of discretionary fat and sugars consumed [7,8].

As a tertiary area of interest we interviewed participants after the trial to examine their tolerability of the MicroN3 foods they ingested. As this was a tertiary measure, we did not use a standardized or validated questionnaire to examine tolerability parameters.

Specific questions included:

(1) Were you able to distinguish the foods you ingested by a fishy odor (Y/N)? If yes, on how many occasions did you notice this phenomenon?

(2) Did the foods you ingest cause you any gastrointestinal distress such as stomach pain, diarrhea, or belching (Y/N)? If yes, on how many occasions did you notice this phenomenon?

(3) Did you notice any fishy aftertaste following the consumption of your breakfast meal (Y/N)? If yes, on how many occasions did you notice this phenomenon?

(4) Did you notice any fishy odor on your breath or with belching (Y/N)? If yes, on how many occasions did you notice this phenomenon?

### Statistical Procedures

We compared all baseline characteristics for demographics and dietary characteristics using a paired t-test. We further examined our participant's baseline dietary intake of N3 fatty acids to the national average of the United States using a one-sample t-test. This was predicated on reports detailing the N3 intake within the United States where total N3 accounts for 1.6 g/d (0.7% of energy intake), 1.4 g/d is plant derived α-linolenic acid (ALA) and 0.1 to 0.2 g/d comes from EPA and DHA [2].

All measures were analyzed for mean within and between group differences and mean delta scores from baseline using a 2 × 2 ANCOA for time (pre/post), group (treatment/control) and time-by-treatment interactions. Tukey post-hoc analyses of statistically significant interactions were used to determine treatment differences at an alpha level of P ≤ 0.05. We examined food tolerability indices using a Chi-Square analysis.

## Results

We observed no significant differences for age (25.4 ± 6.6 y), BMI (25.2 ± 1.4 kg/m^2^), weight (72.9 ± 4.9 kg), or plasma lipids. We have presented the dietary characteristics of our study cohort in Table 1. Overall, we did not observe any statistical difference of the dietary macronutrient composition between treatment groups at baseline or following treatment with the exception of the N3 given to the treated participants. In comparison to reports on national averages, we observed no significant differences between our current cohort and previous reports detailing the N3 intake of those individuals residing the United States.

**Table 1:** Dietary characteristics of study participants

	**Placebo (n = 10)**		**MicroN3 (n = 10)**	
	Mean	SE	Mean	SE
Energy (MJ)	6.74	0.7	6.36	0.6
Protein (g)	73.2	4.4	68	4.4
Carbohydrate (g)	198.8	25.4	186.3	25.4
Total Fat (g)	72.1	4.8	65.1	4.8
Sat Fat (g)	19.5	2.0	18.2	2.0
MUFA (g)	22.9	2.3	21.2	2.3
PUFA (g)	14.9	1.7	11.5	1.7
α-Linoleic (g)	13.1	1.5	12.5	1.5
α-Linolenic (g)	1.4	0.2	1.3	0.2
Arachadonic (mg)	10.1	0.3	10.1	0.3
EPA (mg)	10.1	0.3	10.1	0.3
DHA (mg)	10.1	0.2	10.1	0.2
Cholesterol (mg)	215	37.5	202.9	37.5
Fiber (g)	18.7	3.5	16.7	3.5
Alcohol (g)	7.2	1.7	7.6	1.7

As part of their treatment, the MicroN3 treated group increased their daily intake of EPA/DHA derived N3 by 450–550 mg/d. Following treatment with MicroN3 foods, our statistical analysis showed a significant elevation in mean plasma DHA (P < 0.05) and reduction in triacylglycerols within the treatment group (P < 0.05; Table 2). When expressed as mean delta scores, both the increase in DHA and decrease in triacylglycerols were significantly different from placebo (P < 0.05). While plasma EPA showed a trend to increase in the treatment group, there was no statistical difference noted between the treatment and the placebo group (P = 0.08). Lastly, the results of our tertiary analysis showed no difference between either treatment group, nor no occurrence of questioned effects for any of our interview questions. In essence, our intervention showed no occurrences of being able to identify MicroN3 foods via fish odor from food, gastrointestinal distress, fishy aftertaste or fish odor on the participant's breath.

**Table 2:** Lipid and plasma fatty acid characteristics of the study participants

LIPID PROFILE		Pre-treatment	Post-treatment
Total-C (mmol/L)	Control	5.02 ± 0.2	5.06 ± 0.2
	Treatment	4.22 ± 2.3	4.21 ± 2.2
LDL-C (mmol/L)	Control	3.13 ± 0.2	3.10 ± 0.2
	Treatment	2.42 ± 2.2	2.44 ± 2.3
HDL-C (mmol/L)	Control	1.39 ± 0.1	1.46 ± 0.1
	Treatment	1.34 ± 0.6	1.35 ± 0.7
VLDL-C (mmol/L)	Control	0.50 ± 0.0	0.51 ± 0.1
	Treatment	0.46 ± 0.7	0.42 ± 0.6
Triacylglycerols (mmol/L)^a^	Control	1.01 ± 0.1	1.10 ± 0.3
	Treatment	1.02 ± 0.2	0.91 ± 0.1 ^b, c^
FATTY ACID PROFILE		Pre-treatment	Post-treatment
ALA (*u*mol/L)	Control	22.61 ± 3.4	20.22 ± 2.1
	Treatment	23.18 ± 2.3	19.74 ± 1.7
AA (*u*mol/L)	Control	670.74 ± 60.1	696.77 ± 87.1
	Treatment	599.91 ± 33.9	613.12 ± 27.0
DHA (*u*mol/L)^a^	Control	83.23 ± 10.3	103.23 ± 15.0
	Treatment	91.18 ± 9.7	125.58 ± 11.9 ^b, c^
EPA (*u*mol/L)	Control	22.49 ± 3.4	20.59 ± 6.8
	Treatment	17.93 ± 3.1	20.77 ± 2.9

^a ^Significant overall group × time ANCOVA statistical effect (P < 0.01)

^b ^Represents a significant within group statistical effect (P < 0.05)

^c ^Represents a significant change score different than control (P < 0.05)

Total-C (Total cholesterol), LDL-C (low density cholesterol, HDL-C (high density cholesterol), VLDL (very low density cholesterol)

ALA (alpha-linolenic acid), AA (arachadonic acid), DHA (docosahexaenoic acid), EPA (eicosapentaenoic acid)

## Discussion

The primary findings of our current pilot study show that MicroN3 fortified foods can increase plasma N3 concentrations, while positively modulating triacylglycerols within 2 weeks in a population who would be considered to have normal triacylglycerols concentrations. This latter effect on triacylglycerols is of particular interest as studies showing a reduction in triacylglycerols typically range between 2–4 g of N3 ingestion per day [9]. More recent studies, however, have shown attenuated postprandial triacylglycerols with as little as 1 g/d with chronic administration [10]. The results of our study are appealing as the cohort we examined represents a population similar to the United States national average and the foods ingested were well tolerated.

Collectively, higher N3 consumption has the potential to positively affect many heath issues such as pregnancy, cognitive development and learning in infants and children, visual development, immune and inflammatory responses, rheumatoid arthritis, ulcerative colitis, Crohn disease, eczema, asthma, and type 1 diabetes, metabolic syndrome, type 2 diabetes, obesity, cardiovascular disease and lipid metabolism, neurologic degeneration and mental health and mood disorders [11,12]. Moreover, the U.S. Food and Drug Administration has given a qualified health claim status to EPA and DHA N3 fatty acids, stating that supportive but not conclusive research shows that consumption of EPA and DHA may reduce the risk of coronary heart disease [13].

A fundamental difficulty surrounding the recommendation and ingestion of N3 fatty acids containing high quantities of EPA and DHA is the observation that the highest concentrations of these fatty acids are found in cold water fish [14]. Unfortunately, many individuals are resistant to consuming fish for a variety of reasons including taste, gastrointestinal distress and fish odor [2]. As an alternative to fish ingestion or fish oil supplementation, several studies have examined the effectiveness of ingesting N3 from plant sources, such as flax seed. However, it has been reported that conversion of ALA to EPA and further to DHA in humans is limited, but varies with individuals [15]. For example, it has been reported that women have higher ALA conversion efficiency than men and that conversion is greater than expected in non fish-eating vegetarians and non fish-eating meat-eaters than in fish-eaters [16]. Though the use of N3 fatty acids derived from ALA should not be dissuaded, the effectiveness of longer chain are clearly more effective with regard to efficacy. A major strength of our current pilot study is the suggestion that the incorporation of N3 into common foods shows promise given the increase in plasma DHA, modest lowering of triacylglycerols and lack of side effects reported with MicroN3 food ingestion.

During the study, we were able to deliver 450–550 mg of EPA/DHA or half the dosage recommended by the AHA for patients with documented CHD, and one fourth the dose recommended for individuals with elevated triacylglycerols in one meal [2]. Perhaps one of the most salient findings from this study is that MicroN3 food technology will allow individuals to incorporate N3 more easily into their regular diet. Thus, it is feasible that N3 rich foods can be incorporated into a variety of eating patterns that may be associated with an individual's socioeconomic status, ethnicity, and corresponding food preferences. Though we feel that future investigations into the effects of MicroN3 foods at higher doses, for longer study durations, and with more robust markers of CHD are of merit, the true promise of this technology lies in the potential to deliver long chain N3 fatty acids to individuals not accustomed to nor wanting to ingest fish or fish oil supplements.

We realize that certain limitations can be applied to our current study. First, the sample size was small and that our intervention was relatively short. These two factors most certainly influenced a more accurate portrayal of the biodistribution of the N3 used in our intervention. This is an important factor to consider for future trials using MicroN3 foods as the fraction of N3 (i.e. EPA or DHA) has specific characteristics for dietary interventions. For example, DHA in tissues is particularly abundant in neural and retinal tissue. Further, dietary DHA results in a dose dependent, saturable increase in plasma DHA concentrations accompanied by modest increases in EPA concentrations. Likewise, EPA concentrations increase in response to dietary EPA intake with little increase in DHA concentrations. These same observations are also present for tissue concentrations [17,18]. Conversely, a potential benefit of the MicroN3 technology is that it may allow specific N3 combinations aimed at health specific needs. A second limitation to our study is that we did not record a follow-up food frequency questionnaire. Given that this was only a 2 week pilot trial we are of the opinion that it is unlikely that participants changed their diet in such manner so as alter their overall eating pattern. However, we cannot discount the possibility. Lastly, we feel that our study would have benefited from examining the erythrocytes for N3 concentration.

The strength of our pilot study is that it confirms our hypothesis that foods fortified with MicroN3 can serve as an effective vehicle for the delivery of N3 fatty acids in young, healthy, active participants. Furthermore, the use of such a technology should enable both health care practitioners and consumers alike to make N3 ingestion a part of their normal lifestyle without significantly altering preferred food choices or incorporating a dietary regimen requiring the ingestion of supplement capsules. Our study also demonstrated that a large volume of N3 is easily administered with the alteration of just one daily meal; in our case, a breakfast meal. Therefore, it is not unreasonable to postulate that minor alterations in other daily meals or the augmentation of a capsular supplement routine are well within the grasp of most individuals.

## Conclusion

We conclude that this new food technology shows promise for the development of functional foods capable of improving health care outcomes related to N3 ingestion.

## Competing interests

The authors declare that they have no competing interests.

## Authors' contributions

CPE designed this study and was responsible for all data analysis and the primary writing of this manuscript.

MKH prepared all intervention meals and assisted with the writing of this manuscript.

MM assisted in meal preparation and was responsible for the recruiting and scheduling of study participants.

CRM assisted with data management, analysis and manuscript preparation.

RMD and JAB were responsible for the analysis of all fatty acids.

TSC was the medical director for this trial and assisted in manuscript preparation.
